# Synbiotic supplementation ameliorates anxiety and myocardial ischaemia–reperfusion injury in hyperglycaemic rats by modulating gut microbiota

**DOI:** 10.1113/EP092052

**Published:** 2024-09-12

**Authors:** Erman Caner Bulut, Deniz Erol Kutucu, Savaş Üstünova, Mehmet Ağırbaşlı, Huri Dedeakayoğulları, Çağatay Tarhan, Ayşegül Kapucu, Berrak Ç. Yeğen, Cihan Demirci Tansel, Ebru Gürel Gürevin

**Affiliations:** ^1^ Department of Biology, Institute of Graduate Studies in Sciences Istanbul University Istanbul Turkey; ^2^ Department of Biology, Faculty of Science Istanbul University Istanbul Turkey; ^3^ Department of Physiology, School of Medicine Bezmialem Vakıf University Istanbul Turkey; ^4^ Department of Cardiology, School of Medicine Istanbul Medeniyet University Istanbul Turkey; ^5^ Department of Medical Biochemistry, Faculty of Medicine Biruni University Istanbul Turkey; ^6^ Department of Molecular Biology and Genetics, Faculty of Science Istanbul University Istanbul Turkey; ^7^ Department of Physiology, School of Medicine Marmara University Istanbul Turkey

**Keywords:** anxiety, gut dysbiosis, high fat and high carbohydrate diet, myocardial ischaemia–reperfusion, streptozotocin, synbiotic

## Abstract

Hyperglycaemia, hyperlipidaemia, hypertension and obesity are the main risk factors affecting the development and prognosis of ischaemic heart disease, which is still an important cause of death today. In our study, male Sprague–Dawley rats were fed either a standard diet (SD) or a high fat and high carbohydrate diet (HF‐HCD) for 8 weeks and streptozotocin (STZ) was injected at the seventh week of the feeding period. In one set of rats, a mixture of a prebiotic and probiotics (synbiotic, SYN) was administered by gavage starting from the beginning of the feeding period. Experimental myocardial ischaemia–reperfusion (30 min/60 min) was induced at the end of 8 weeks. Hyperglycaemia, hypertension and increased serum low‐density lipoprotein levels occurred in SD‐ and HF‐HCD‐fed and STZ‐treated rats followed for 8 weeks. Increased density of the *Proteobacteria* phylum was observed in rats with increased blood glucose levels, indicating intestinal dysbiosis. The severity of cardiac damage was highest in the dysbiotic HF‐HCD‐fed hyperglycaemic rats, which was evident with increased serum creatine kinase‐MB (CK‐MB), cardiac troponin I (cTnI), tumour necrosis factor‐α, and interleukin‐6 levels, along with a decrease in ST‐segment resolution index. SYN supplementation to either a normal or a high‐fat high‐carbohydrate diet improved gut dysbiosis, reduced anxiety, decreased CK‐MB and cTnI levels, and alleviated myocardial ischaemia–reperfusion injury in hyperglycaemic rats.

## Abstract (highlights)

- **What is the central question of this study?**
  Could synbiotic supplementation have ameliorative effects on myocardial damage and anxiety by regulating gut microbiota? If so, how is the ameliorative effect of correcting gut microbiome dysbiosis manifested?
- **What is the main finding and its importance?**
  Synbiotic supplementation decreased the abundance of the *Proteobacteria* phylum, which is an indicator of intestinal dysbiosis, inhibited the formation of pro‐inflammatory cytokines, and improved myocardial damage and anxiety, suggesting that it may form a new approach to improving myocardial damage and anxiety.

## 1 INTRODUCTION

Despite improved invasive and non‐invasive treatments, cardiovascular disease (CVD) is still a major cause of death, responsible for 30% of all deaths (Arshad et al., 2016). Degenerative changes in coronary arteries or blood vessels, which prevent the delivery of oxygen and nutrients to the myocardium, play a major role in the development of CVDs that include ischaemic heart disease, atherosclerosis, hypertension and heart failure (Frank et al., 2012). The main strategy to prevent the ischaemic damage or to reduce its severity is to identify and eliminate the risk factors. High‐fat and high‐carbohydrate diet (HF‐HCD), dyslipidaemia, diabetes, hypertension, and obesity are common risk factors in the development of cardiac ischaemia (Ferdinandy et al., 2007). Recent studies have reported that HF‐HCD has an enhancing effect on risk factors by altering the density of the intestinal microbial phyla (Pozzo et al., 2016).

The gut is host to more than 50 phyla (Salamat et al., 2024), the most dominant of which are *Firmicutes*, *Bacteroidetes*, *Proteobacteria* and *Actinobacteria* (Wan et al., 2023; Yang et al., 2024). The density of a phylum can change under different conditions, and changes in the type of diet are one of the important factors that can affect the density of a phylum. HF‐HCD is known to cause dysbiosis by increasing the density of the *Proteobacteria* phylum (Imdad et al., 2024). An increase in this phylum has been associated with obesity, hyperglycaemia, type 2 diabetes (T2DM) and prediabetes (Peters et al., 2018; Rizzatti et al., 2017). Another study in obese rats revealed an increase in *Firmicutes* and *Proteobacteria* along with a decrease in *Bacteroidetes* levels (Yue et al., 2024). In a study on germ‐free mice fed with HF‐HCD, animals that were not initially obese became less resistant to obesity and gained weight when they were administered *Enterobacter* (Yan et al., 2016). A study on the development of atherosclerotic plaque emphasized the presence of a high concentration of bacteria from the *Proteobacteria* phylum in the plaques (Ziganshina et al., 2016). It has also been noted that an increase in the density of the *Proteobacteria* phylum in the gut leads to a pro‐inflammatory response and increases the level of obesity and hyperlipidaemia (Harris et al., 2012; Politi et al., 2023). Studies have shown that changes in microbial composition also have a significant impact on cognitive health (Noble et al., 2017).

Synbiotics are supplements that combine probiotic and prebiotic ingredients. Without prebiotics, probiotics are less resistant to oxygen, low pH levels and temperature (Sekhon & Jairath, 2010). Research has shown that using prebiotics and probiotics together increases the growth of beneficial bacteria (Pranckutė et al., 2016). The preventive advantages of synbiotics against many diseases have been investigated in several studies. Synbiotics positively affect the control of intestinal metabolic activity and the maintenance of intestinal microbiological structure. Since dysbiosis was recently described as a risk factor for CVD, synbiotics are expected to serve as a treatment for CVD (Naseri et al., 2022).

In light of the above‐mentioned studies, we aimed to explore the possible beneficial effects of synbiotic supplementation on myocardial ischaemic injury and anxiety in hyperglycaemic rats that were previously fed with either a standard diet (SD) or a HF‐HCD.

## 2 METHODS

### 2.1 Ethical approval

All experimental protocols were evaluated and approved by Bezmialem Vakıf University Animal Experiments Local Ethics Committee (28.09.2020; BVÜ‐HADYEK‐2020/138). The experiments were carried out ethically in accordance with the EU Animal Experiments Directive 2010/63/EU.

The myocardial ischaemia–reperfusion procedure in rats was applied after deep anaesthesia was achieved. At the end of this procedure, blood samples were collected from the right atrium with a syringe. After cardiac puncture, euthanasia was accomplished with the arrest of the heart.

### 2.2 Animals and experimental design

The study was conducted in male Sprague–Dawley rats (40–50 g, 21 days old) supplied by the Bezmialem Vakıf University Experimental Animals Application and Research Centre. The rats were kept in a room with controlled temperature (21 ± 2°C), humidity (65–70%) and a 12 h light/12 h dark cycle and were given tap water and food ad libitum.

**FIGURE 1:**
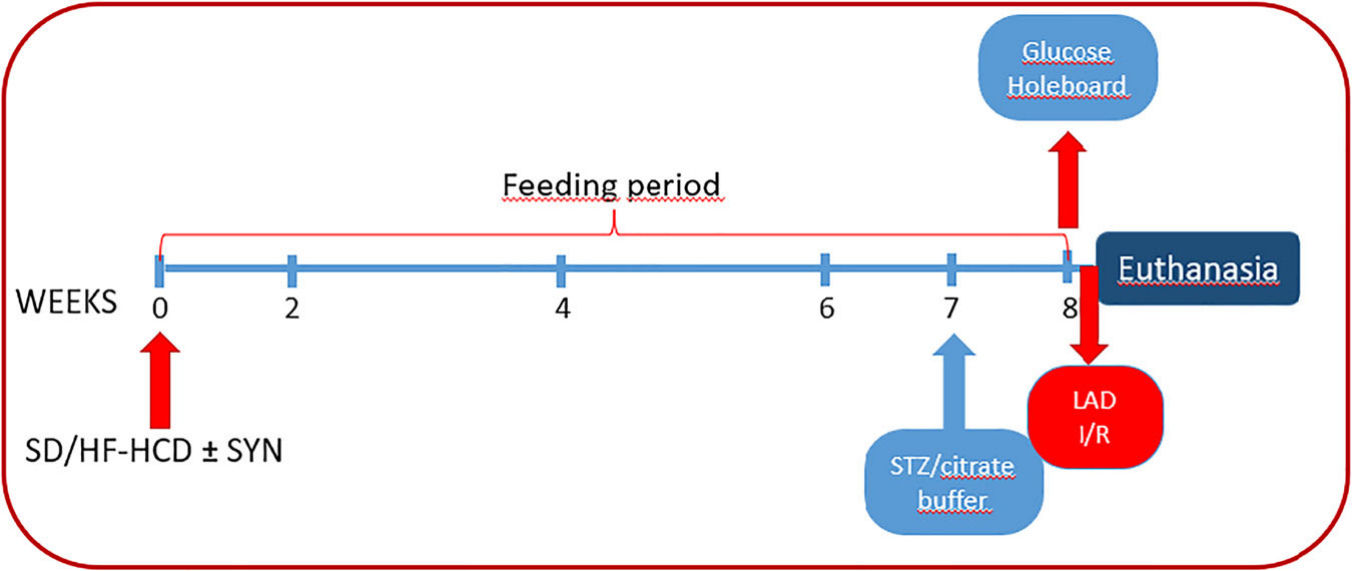
Flowchart of the experiment. Rats were divided into five different groups with eight rats in each group: (1) citrate buffer‐injected control rats on a standard diet (SD), (2) streptozotocin (STZ)‐injected hyperglycaemic rats on SD, (3) hyperglycaemic rats on SD supplemented with synbiotics (SYN), (4) hyperglycaemic rats on high‐fat high‐carbohydrate diet (HF‐HCD), and (5) hyperglycaemic rats on HF‐HCD supplemented with SYN. Rats were fed SD or HF‐HCD for 8 weeks.

Starting by the post‐natal day 21, the rats were randomly divided into two groups and were fed either a SD (Altromin 1310, Lage, Germany; *n* = 24) or a HF‐HCD (Arden Western Diet, Ankara, Türkiye; *n* = 16) for 8 weeks after measurement of baseline body weight (Figure 1). During this 8‐week period, two subgroups of rats on either SD or HF‐HCD were administered daily with a synbiotic supplement (SYN) at a dose of 2400 mg/kg/day (1500 mg/kg/day probiotics and 900 mg/kg/day prebiotic in 2 mL saline) by orogastric gavage SYN used in our study (NTBIOTIC Synbiotic, Assos Pharma, Istanbul, Türkiye) consisting of probiotics (250 mg; *Lactobacillus acidophilus*, *Lactobacillus bulgaricus*, *Streptococcus thermophilus*, *Bifidobacterium longum*, *Bifidobacterium bifidum*, *Bifidobacterium infantis*) and a prebiotic (150 mg inulin) (Basturk et al., 2016; Mazloom et al., 2013; Yang et al., 2005). At the end of the 7‐week feeding period, rats were injected intraperitoneally (i.p.) with a low dose of streptozotocin (STZ, 30 mg/kg; *n* = 32) to induce hyperglycaemia (Cheng et al., 2018), while a subgroup of SD‐fed rats was given 0.1 M citrate buffer of pH 4.5 (normoglycaemic control group) (Kaur, 2012). STZ (Sigma‐Aldrich, St Louis, MO, USA) was dissolved in 0.1 M cold citrate buffer of pH 4.5 (Sigma‐Aldrich) (Motyl & McCabe, 2009).

On the last day of the experiment (56th day), body weights were measured, and a hole‐board test was used to determine the anxiety levels of rats. All experimental procedures and blood withdrawals were performed at the same time of day to minimise environmental variables. Blood samples were taken from the jugular vein for the measurement of blood glucose levels using a glucometer (Contour Plus, Ascensia Diabetes Care Holdings, Basel, Switzerland). Rats were placed in a position suitable for blood sampling by grasping the loose skin on their backs. A 27‐calibre needle was inserted into the left jugular vein through the pectoral muscle just below the junction of sternum and clavicle. Using a 1‐mL syringe, 10 µL of blood was slowly withdrawn, avoiding collapse of veins. As soon as blood was drawn, the site was gently pressed to stop bleeding (Parasuraman et al., 2010).

Rats were then anaesthetized with pentobarbital (50 mg/kg i.p.; Pental Sodium, IE Ulagay, Istanbul, Türkiye), and following tracheostomy, right carotid artery cannulation and electrocardiographic recording (ECG) were performed for haemodynamic evaluation. Afterwards, the chest of the rats was opened with a left parasternal incision and an experimental myocardial ischaemia–reperfusion (I/R) model, with 30‐min ischaemia and 60‐min reperfusion, was applied (Bai et al., 2021). After 60 min of reperfusion, the rats were euthanized by right atrial puncture to obtain blood samples, while faecal samples were collected from the terminal caecum of all rats. To preserve the presence and concentration of bacterial DNA in faeces, samples were stored at −80°C until analysed by q‐PCR.

### 2.3 Evaluation of anxiety

To evaluate the effect of HF‐HCD, hyperglycaemia and/or SYN supplementation on the anxiety levels of rats before they were induced with cardiac ischaemia, a hole‐board test was performed in all rats at 30 min before blood was taken from the jugular vein for blood glucose level measurement. The hole‐board apparatus (40 cm × 40 cm) consisted of 16 equally spaced holes, each with a diameter of 3.8 cm, which is commonly used to determine anxiety in rats (Viveros et al., 2005). The motor activity of the rat positioned in the middle of the apparatus was recorded for 5 min. The video recordings of the rats were watched and counts of hole‐dipping and freezing time were reported by an observer blinded to the experimental groups. Since anxiety reduces natural exploratory behaviour in rats, a decrease in the number of head‐dippings into the hole, and an increase in the duration of freezing were considered as enhanced anxiety.

### 2.4 Anaesthesia and carotid artery cannulation

Following the anxiety test and blood glucose measurements, rats were anaesthetized with pentobarbital (50 mg/kg i.p.) before the surgical procedure. The depth of anaesthesia in rats was checked every 10 min during the experimental procedure by limb withdrawal and cornea reflexes, and when needed, pentobarbital at an additional dose of 5 mg/kg was administered to maintain deep anaesthesia.

For tracheostomy and carotid artery cannulation, an incision (1.5 cm) was made on the neck to access the platysma muscle, which was carefully exposed with a non‐traumatic forceps to reach trachea. An intubation tube (8FR) was inserted through a small incision made on the trachea, and the tube was secured by a silk suture placed around the trachea. After the chest wall was opened, the end of the tracheal tube was connected to the ventilator to apply mechanical ventilation.

The carotid artery was accessed through the same opening, and surrounding connective tissues were removed carefully by avoiding the stimulation of vagus nerve. Then an incision was done on the carotid artery to insert a 2‐mm cannula filled with heparinized saline (0.5 IU/mL) and was connected to a pressure transducer. Following a 5‐min stabilisation period, arterial pressure was recorded for an additional 5 min until the chest cavity was opened for the induction of myocardial ischaemia–reperfusion. Data were recorded on a data acquisition and interpretation system (PowerLab ML870B2, ADInstruments, Bella Vista, NSW, Australia) for later assessment.

### 2.5 Induction of myocardial ischaemia–reperfusion

**FIGURE 2:**
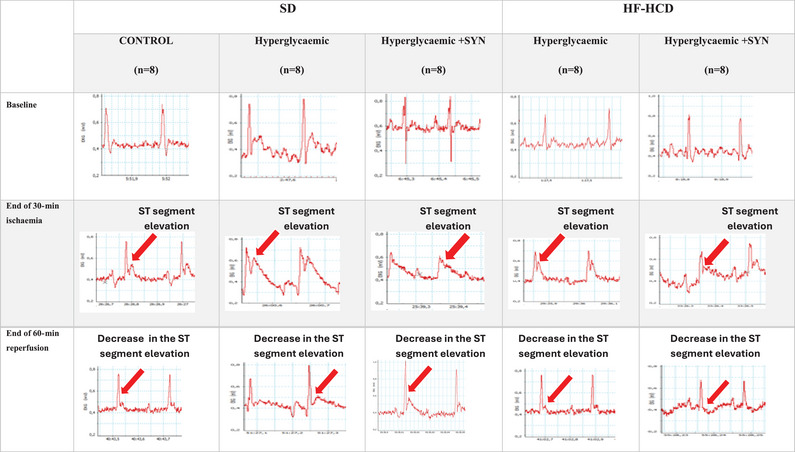
Representative electrocardiographic (ECG) recordings of all groups at baseline, end of 30‐min ischaemia and end of 60‐min reperfusion. The vertical axis of the ECG trace shows voltage (mV). The horizontal axis of the ECG trace shows time (0.2 s). The red arrow shows the ST segment elevation during ischaemia and the decrease in ST segment elevation during reperfusion.

After controlling the depth of anaesthesia, needle electrodes were placed under the skin of peripheral extremities to obtain lead II recording. Following the stabilization of ECG and blood pressure measurements, a 3–4 cm transverse incision was made between the left third and fourth ribs, and it was enlarged with a retractor and the chest cavity was opened. The rat was then ventilated via a mechanical ventilator (Rodent Ventilator Model 683, Harvard Apparatus, Holliston, MA, USA) to provide a tidal volume of 0.9 mL and a respiratory rate of approximately 80 breaths per minute. At the end of this procedure, the heart was exposed at the incision site and the pericardium was dissected out with non‐traumatic forceps. A suture (7‐0 silk; Ethicon, Raritan, NJ, USA) was then positioned around the left anterior descending coronary artery (LAD), below the left atrial appendage, close to its origin. Both ends of the suture were inserted through a 2‐mm PVC tube to form a ligature. To induce myocardial ischaemia, tension was applied to the ligature and LAD was occluded for 30 min. The occurrence of ischaemia was evidenced with epicardial cyanosis, hyperaemia and ST segment elevation (Figure 2). At the end of 30 min, the ligature was relieved to reduce tension and the 60‐min reperfusion process was started.

### 2.6 Analysis of ST segment changes and blood pressure

ECG recordings obtained before ischaemia, during ischaemia and following reperfusion were retrospectively evaluated using a calliper (at 60 ms beyond the J point) by a cardiologist blinded to the experimental groups. The maximum ST segment elevation in the ECG recording obtained before ischaemia was used as a reference. An increase in ST segment elevation during ischaemia with respect to the reference indicated the success of ischaemia. During reperfusion, a more than 50% decrease in the maximum ST segment elevation that was recorded at 30 min of ischaemia showed that reperfusion was successful. For haemodynamic evaluation, ST dissolution index (STRI) was calculated by dividing the ST segment elevation (mm) recorded at 30 min of ischaemia by the ST segment elevation (mm) at 60 min of reperfusion. Sample ECG tracings used for STRI calculation are presented in Figure 2.

### 2.7 Blood assays

At the end of the 60‐min reperfusion period, blood samples were obtained by cardiac puncture from the right atrium and rats were then euthanized. Serum was obtained from the blood samples using a centrifuge (NF‐200, Nüve, Turkey) at 1800 *g* for 15 min, and stored at −80°C. Before biochemical analyses, samples were defrosted in a laboratory‐type cooler (HYC‐610, Haier, Qingdao, China) at +4°C for 8 h. After dissolution, the samples were analysed by enzyme‐linked immunosorbent assay (ELISA). Serum samples were analysed for low density lipoprotein (LDL), high density lipoprotein (HDL), cardiac troponin I (cTnI), creatine kinase‐MB (CK‐MB), tumour necrosis factor‐α (TNF‐α), interleukin‐6 (IL‐6), and corticosterone (Abbkine Scientific Co., Ltd, Atlanta, GA, USA). The manufacturer's instructions were followed during the analyses. Reactions were analysed by ELISA Microplate Reader (BK‐EL10C, Biobase, Shandong, China). Results are presented in milligrams per decilitre.

### 2.8 Microbiological analysis of faecal samples

Faecal samples collected from the experimental groups were stored at −80°C, and DNA extraction was performed using a DNA isolation kit (PCR Biosystems Inc., Wayne, PA, USA). Analyses to evaluate the concentrations of bacterial DNA were performed using Real Time PCR Thermal Cycler (CFX 96, Bio‐Rad Laboratories, Paris, France) with *Proteobacteria*, *Bacteroidetes*, *Firmicutes* and *Actinobacteria* phylum specific primers (Bacchetti De Gregoris et al., 2011) and qPCRBIO SyGreen Mix kit (PCR Biosystems) according to the manufacturer's instructions.

### 2.9 Statistical analyses

Statistical analyses were performed with GraphPad Prism 8.3.0 (GraphPad Software Inc., San Diego, CA, USA). The data were stated as means ± standard deviation (SD) and were analysed by one‐way ANOVA followed by a post‐hoc Tukey test. *P* < 0.05 values were accepted as significant. Correlations between cardiometabolic risk factors, markers of cardiac damage, inflammation and anxiety, and intestinal bacterial phylum density were analysed by Pearson's correlation test.

## 3 RESULTS

### 3.1 Effects of synbiotic supplementation on body weight and metabolic parameters

When compared with normoglycaemic‐SD‐fed control group, body weights were increased in both HF‐HCD‐fed groups supplemented with or without SYN (*P *< 0.0001, *n* = 16; Table 1). On the other hand, the body weights of the HF‐HCD‐fed group that had received SYN were significantly reduced compared to the HF‐HCD group without SYN intake (*P *= 0.0028; Table 1). The difference between the final weight measurements and the initial body weights of the rats shows the change in body weight. The body weight change in HF‐HCD‐fed hyperglycaemic rats with or without SYN supplementation increased significantly compared to the control group (*P *< 0.0001). Change in body weight was significantly decreased in the HF‐HCD‐fed group receiving SYN compared to the HF‐HCD group not receiving SYN (*P *= 0.0040).

**TABLE 1:** Differences in body weights, blood glucose and serum lipid levels between groups before LAD ligation induced myocardial I/R period.

	Standard diet						High fat and high carbohydrate diet				
	Control (*n* = 8)	HG (*n* = 8)	*P* (Control versus HG)	HG +SYN (*n* = 8)	*P* (Control versus HG+SYN)	*P* (HG versus HG+SYN)	HG (*n* = 8)	*P* (Control versus HG)	HG +SYN (*n* = 8)	*P* (Control versus HG+SYN)	*P* (HG versus HG+SYN)
Body weight (g)	362.0 ± 22.46	358.50 ± 21.37	0.779	368.50 ± 12.03	0.929	0.559	481.50 ± 25.36	<0.0001	440.00 ± 18.65	<0.0001	0.0028
Change in weight (g)	316.6 ± 22.46	313.1 ± 21.37	0.929	323.1 ± 12.03	0.779	0.559	436.1 ± 25.36	<0.0001	395.3 ± 18.65	<0.0001	0.0040
Blood glucose level (mg/dl)	97.20 ± 7.49	257.30 ± 13.82	<0.0001	144.87 ± 15.20	<0.0001	<0.0001	163.20 ± 5.70	<0.0001	153.00 ± 4.03	<0.0001	0.0049
Serum HDL level (mg/dl)	37.55 ± 0.804	34.51 ± 2.17	0.017	37.10 ± 1.67	0.987	0.0009	33.89 ± 1.99	0.002	34.64 ± 2.17	0.024	0.923
Serum LDL level (mg/dl)	96.89 ± 11.44	126.90 ± 11.22	<0.0001	115.60 ± 9.89	0.009	0.223	119.80 ± 13.09	0.0036	113.80 ± 5.27	0.022	0.784

*Note*: Data are given as means ± SD and each group consists of eight rats. Abbreviations: HG, hyperglycaemic; HG+SYN, hyperglycaemic with synbiotic supplement.

Blood glucose levels were increased significantly in all STZ‐injected groups that were fed either the SD or the HF‐HCD (*P *< 0.0001, *n* = 16; Table 1), showing that STZ injection had resulted in hyperglycaemia. On the other hand, feeding with SYN in the SD and HF‐HCD groups significantly reduced the blood glucose level compared to the SD and HF‐HCD fed group without SYN supplementation, respectively (SD: *P *< 0.0001 and HF‐HCD: *P *= 0.0049). Serum HDL levels were significantly reduced in hyperglycaemic rats independent of diet type (SD: *P* = 0.017 and HF‐HCD: *P* = 0.002). SYN supplementation significantly increased serum HDL levels in SD‐fed hyperglycaemic rats (*P* = 0.009). Serum LDL levels were significantly increased in the hyperglycaemic rats that were not supplied with SYN (SD: *P = *0.0003 and HF‐HCD: *P = *0.0036). However, when hyperglycaemic groups on either diet were supplemented with SYN, their LDL levels were not different from that of the normoglycaemic control group.

### 3.2 Effect of synbiotic supplementation on anxiety level

**FIGURE 3:**
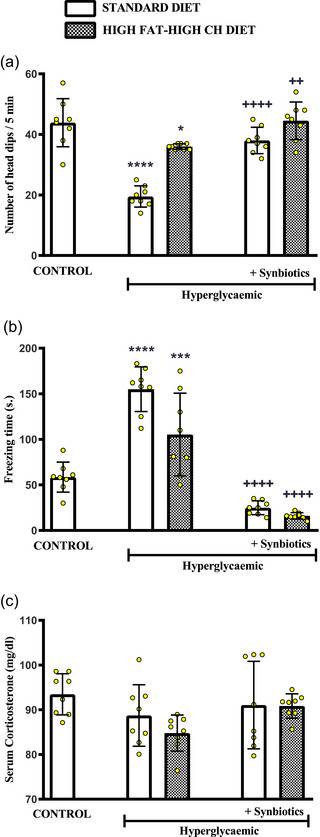
Anxiety levels of all groups before myocardial I/R induced by LAD ligation. (a) Number of head dips/5 min, (b) freezing time (s), and (c) serum corticosterone levels (mg/dl). Data are given as means ± SD and each group consists of eight rats. ^*^P = 0.0123, ^***^P = 0.0007, ^****^P < 0.0001 compared to control group; *^++^P *= 0.0054, *^++++^P *< 0.0001 compared to respective hyperglycaemic group without synbiotic supplementation.

Anxiety levels of the rats before the induction of cardiac I/R injury were evaluated by the activity of the rats (*n* = 40) in the hole‐board apparatus. When compared with the normoglycaemic control group, the number of head‐dippings within 5 min was significantly decreased in hyperglycaemic rats on either diet (SD: *P *< 0.0001 and HF‐HCD: *P *= 0.0123; Figure 3a), which suggests an increased level of anxiety. On the other hand, addition of SYN to either the SD (*P *< 0.0001) or the HF‐HCD (*P *= 0.0054) depressed the anxiety levels of rats, which was evidenced by the reversal of the hyperglycaemia‐induced reduction in head‐dipping. In parallel to these results, freezing time measured in both groups of hyperglycaemic rats with either diet was increased with respect to control group (SD: *P *< 0.0001 and HF‐HCD: *P *= 0.0007; Figure 3b), while SYN supplementation returned freezing time to control levels in both SD and HF‐HCD‐fed rats (*P *< 0.0001; Figure 3b). Despite the changes in anxiety levels as measured by hole‐board, serum corticosterone levels of the groups were similar (Figure 3c).

### 3.3 Effects of synbiotic supplementation on serum cardiac markers and haemodynamic parameters

**FIGURE 4:**
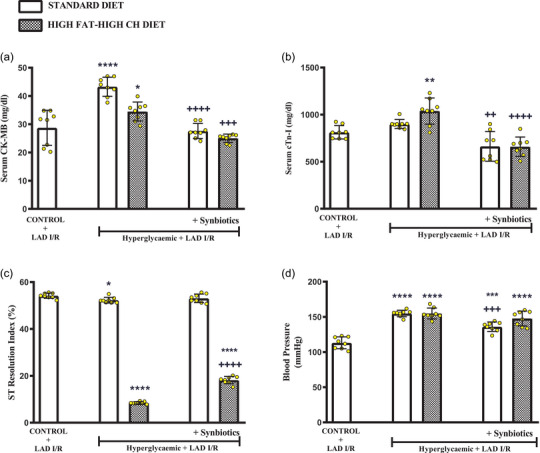
In all rats, changes in serum markers of cardiac injury under myocardial I/R conditions induced by LAD ligation. (a) Serum CK‐MB levels (mg/dl), (b) serum CTn‐I levels (mg/dl), (c) ST Resolution Index (%), and (d) blood pressure (mmHg) levels before myocardial I/R conditions. Data are given as means ± SD and each group consists of eight rats. ^*^P = 0.031 and ^*^ *P *= 0.049, ^**^P = 0.0024, ^***^P = 0.0003, ^****^P < 0.0001 compared to control group; *^++^P *= 0.0012, *^+++^P *= 0.0003, *^++++^P *< 0.0001 compared to respective hyperglycaemic group without synbiotic supplementation.

As an indicator of cardiac ischaemia, serum levels of CK‐MB were significantly increased in cardiac I/R‐induced hyperglycaemic rats on either diet (SD: *P *< 0.0001 and HF‐HCD: *P *= 0.031; Figure 4a). However, addition of SYN to either diet abolished the elevation in CK‐MB levels, which were not different from that of the control group. Serum cTn‐I level, as another indicator of cardiac ischaemia, was not different from that of the control group in the SD‐fed hyperglycaemic I/R group, but cTn‐I was significantly elevated when the I/R‐rats were fed with HF‐HCD (*P *= 0.0024; Figure 4b). On the other hand, supplementation with SYN before cardiac I/R significantly decreased serum cTn‐I level in rats on either diet (SD: *P *= 0.0012 and HF‐HCD: *P *< 0.0001).

Calculated ST resolution index (STRI; %) was slightly decreased in SD‐fed hyperglycaemic rats compared to control group (*P *= 0.049), while the reduction was exaggerated in rats on a HF‐HCD (*P *< 0.0001; Figure 4c). However, STRI was increased in both SD‐ and HF‐HCD‐fed rats that were supplemented with SYN, and this elevation was statistically different in HF‐HCD group as compared to respective group without SYN supplementation (*P *< 0.0001). When compared with that of the control group, BP was increased in all hyperglycaemic I/R‐rats that were fed with either the SD or the HF‐HCD (SD: *P *< 0.0001 and HF‐HCD: *P *< 0.0001; Figure 4d). However, having SYN supplementation for 8 weeks significantly decreased BP in SD‐fed hyperglycaemic rats (*P *= 0.0003), but not in HF‐HCD‐fed rats.

### 3.4 Effect of synbiotic supplementation on serum inflammation markers

**FIGURE 5:**
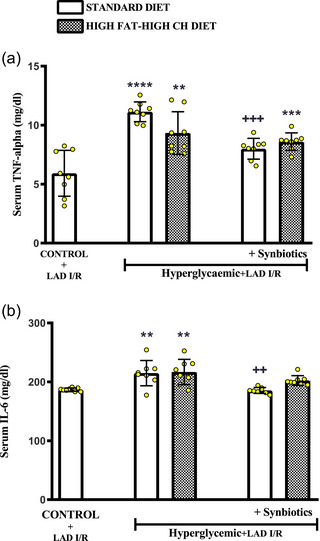
In all rats, changes in serum inflammation markers under myocardial I/R conditions induced by LAD ligation. (a) Serum TNF‐α levels (mg/dl) and (b) serum IL‐6 levels (mg/dl). Data are given as means ± SD and each group consists of 8 rats. ^**^P = 0.0013, ^**^P = 0.0029, and ^**^P = 0.0016, ^***^P = 0.0007, ^****^P < 0.0001 compared to control group; *^++^P = *0.0020, *^+++^P *= 0.0007 compared to respective hyperglycaemic group without synbiotic supplementation.

Serum levels of TNF‐α, a pro‐inflammatory cytokine, were increased significantly in all cardiac I/R‐induced hyperglycaemic rats as compared to control group (SD: *P *< 0.0001 and HF‐HCD: *P *< 0.0013; Figure 5a). while TNF‐a was further elevated in the serum of hyperglycaemic rats fed the SD without SYN supplementation (*P *< 0.0001). On the other hand, IL‐6 level was significantly increased in the serum of I/R‐induced rats that were not supplemented with SYN (SD: *P *= 0.0029 and HF‐HCD: *P *= 0.0016; Figure 5b). Addition of SYN abolished the elevation in serum TNF‐α and IL‐6 levels of I/R‐induced hyperglycaemic rats, but this reduction was statistically significant only in the SD‐fed group (*P *= 0.0020).

### 3.5 Effect of synbiotic supplementation on the density of common bacterial phyla in the gut microbiota

**FIGURE 6:**
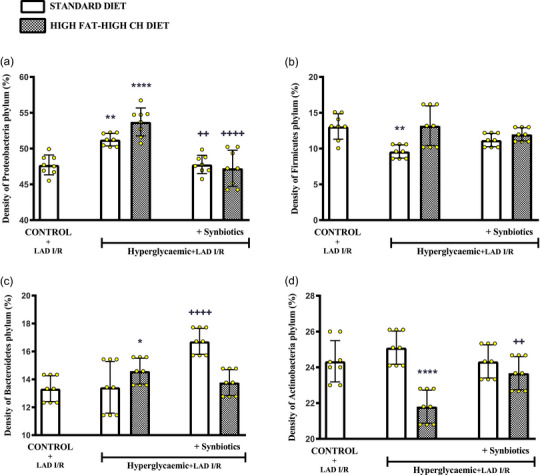
Density (%) of common bacterial phyla in the gut microbiota of all rats. Density of (a) *Proteobacteria* phylum, (b) *Firmicutes* phylum, (c) *Bacteroidetes* phylum, and (d) *Actinobacteria* phylum. Data are given as means ± SD and each group consists of eight rats. ^*^ *P *= 0.035, ^**^P = 0.0018 and ^**^ *P *= 0.0013, ^****^P < 0.0001 compared to control group; *^++^P *< 0.0022 and *^++^P *< 0.0043, *^++++^P *< 0.0001 compared to respective hyperglycaemic group without synbiotic supplementation.

When compared with the control group, the density of *Proteobacteria* phylum in the faecal material was significantly higher in cardiac I/R‐induced hyperglycaemic rats fed with either the SD (*P *= 0.0018) or the HF‐HCD (*P *< 0.0001) (Figure 6a). However, supplementing with SYN decreased the density of *Proteobacteria* (SD: *P *= 0.0022 and HF‐HCD: *P *< 0.0001). On the other hand, the density of *Firmicutes* was reduced in the hyperglycaemic SD‐fed group as compared to control group (*P *= 0.0013; Figure 6b), but *Firmicutes* density in all the other groups was not different from that of the control group. In HF‐HCD‐fed hyperglycaemic rats that underwent cardiac I/R, the density of *Bacteroidetes* was higher (*P *= 0.035; Figure 6c), while *Actinobacteria* density was lower as compared to control group (*P *< 0.0001; Figure 6d). SYN supplementation resulted in an increase in the density of *Bacteroidetes* in SD‐fed hyperglycaemic rats (*P *= 0.0001), while *Actinobacteria* were increased in the HF‐HCD‐fed group supplemented with SYN (*P *= 0.0043).

### 3.6 Correlation between *Proteobacteria* phylum density (%) and blood glucose level, markers of cardiac damage, markers of inflammation and anxiety level

**FIGURE 7:**
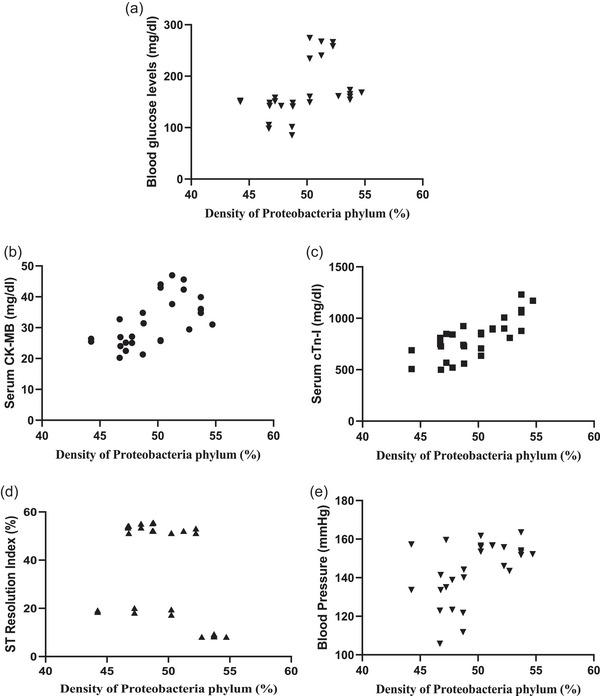
Correlation between *Proteobacteria* phylum density (%) and markers of cardiac damage. Correlation between *Proteobacteria* phylum density (%) and (a) blood glucose levels (*n* = 8, *r* = 0.4302, *P = *0.0223), (b) CK‐MB (*n* = 8, *r *= 0.5955, *P = *0.0008), (c) cTn‐I (*n* = 8, *r* = 0.7606, *P <* 0.0001), (d) ST resolution index (%) (*n* = 8, *r* = −0.3767, *P = *0.0482), and (e) blood pressure (mmHg) (*n* = 8, *r* = 0.5012, *P = *0.0066).

**FIGURE 8:**
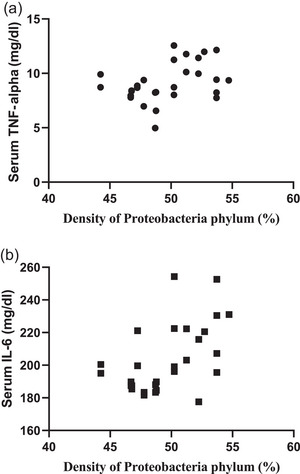
Correlation between *Proteobacteria* phylum density and markers of inflammation. Correlation between *Proteobacteria* phylum density (%) and (a) TNF‐α levels (*n* = 8, *r* = 0.4746, *P = *0.0107) and (b) IL‐6 levels (*n* = 8, *r* = 0.5174, *P = *0.0048).

A significant positive correlation was observed between *Proteobacteria* phylum density and blood glucose level (*r* = 0.4302, *P *= 0.0223), serum CK‐MB level (*r* = 0.5955, *P *= 0.0008), serum cTn‐I level (*r* = 0.7606, *P *< 0.0001) and blood pressure (*r* = 0.5012, *P *= 0.0066; Figure 7), showing increased *Proteobacteria* phylum density as an important cardiometabolic risk factor contributing to increased cardiac injury. Similarly, a positive correlation was present between *Proteobacteria* phylum density and serum TNF‐α (*r* = 0.4746, *P *= 0.0107) and IL‐6 levels (*r* = 0.5174, *P *= 0.0048; Figure 8), which are markers of inflammation. On the other hand, no correlation was observed between the density of other phyla and blood glucose, serum CK‐MB, cTn‐I, TNF‐α, IL‐6 levels or blood pressure (data not shown). A negative correlation was present between *Proteobacteria* phylum density and ST Resolution Index (*r* = −0.3767, *P *= 0.0482; Figure 7d), which indicates the severity of cardiac damage and the extent of necrotic area.

**FIGURE 9:**
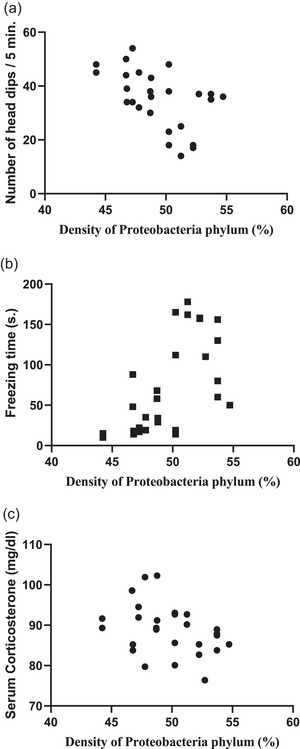
Correlation between *Proteobacteria* phylum density (%) and markers of anxiety. Correlation between *Proteobacteria* phylum density (%) and (a) number of head dips (*n* = 8, *r* = −0.4746, *P = *0.0107), (b) freezing time (*n* = 8, *r* = 0.6143*, P = *0.0005), and (c) serum corticosterone levels (*n* = 8, *r* = −0.3974, *P = *0.0039).

In addition, the correlation between *Proteobacteria* phylum density and anxiety was evaluated and it was observed that there was a negative correlation between *Proteobacteria* phylum density and the number of times the rats looked in the hole (*r* = −0.4746, *P *= 0.0107) accompanied by a positive correlation with freezing time (*r* = 0.6143, *P *= 0.0005; Figure 9), implicating that anxiety level increases with increased *Proteobacteria* phylum. In addition, a negative correlation (*r* = −0.3974, *P *= 0.0039; Figure 9) between *Proteobacteria* phylum density and serum corticosterone levels was observed, supporting the anxiety test results. However, no correlation was observed between the density of other phyla and anxiety test results or corticosterone levels (data not shown).

## 4 DISCUSSION

Obesity, high serum lipid levels and hyperglycaemia, which are the leading CVD risk factors (McLaughlin et al., 2004), contribute to the occurrence of cardiac diseases and intensify the severity of cardiac damage (Pipicz et al., 2018). In our study, STZ‐induced hyperglycaemia and feeding with a high fat and high carbohydrate diet was used to simulate these risk factors (Zhang et al., 2008). In all the rats injected with STZ, hyperglycaemia has developed regardless of diet, while the increased weight gain was observed in HF‐HCD‐fed hyperglycaemic rats, but not in SD‐fed hyperglycaemic rats. However, serum LDL level, which is another critical CVD risk factor, was elevated in SD‐fed hyperglycaemic rats. It has been advocated that cardiovascular dysfunction observed in STZ‐induced diabetes is more closely related to hyperglycaemia and changes in serum lipid profile rather than body weight changes (Schaan et al., 2004). Interestingly, our data also showed that blood glucose levels of HF‐HCD‐fed hyperglycaemic rats were significantly lower than the blood glucose levels of hyperglycaemic rats that were fed with a SD. It was previously reported that increased glucagon released from pancreatic α‐cells exacerbates hyperglycaemia, while high fat added to the diet had a protective effect against hyperglycaemia by producing a hypoglucagonaemic effect (Wang et al., 2015). In addition, an increase in *Proteobacteria* phylum was detected in hyperglycaemic rats fed with either diet, suggesting that the increase in *Proteobacteria* phylum has occurred independent of nutritional differences. In a recent study, it was reported that intestinal dysbiosis in hyperglycaemic patients with impaired serum lipid profiles was evident by the overgrowth of *Proteobacteria* phylum (Oshim et al., 2023). The findings obtained in our study support these data and indicate that STZ‐induced hyperglycaemia promotes the development of intestinal dysbiosis in rats. On the other hand, daily SYN supplementation via its modulatory effects on gut microbiota improved dysbiosis by depressing the increase in *Proteobacteria* density in hyperglycaemic rats fed with either diet. In addition, SYN supplementation prevented body weight gain, decreased blood glucose levels and suppressed the increase in serum LDL levels.

CK‐MB is a sensitive marker for the detection of the ischaemic state, while troponins are more specific markers for the diagnosis of myocardial necrosis (Wu et al., 1996). Accordingly, CK‐MB levels, which indicate I/R‐induced cardiac damage, were increased in SD‐fed hyperglycaemic rats, but the slight elevation in cTnI levels was not statistically significant, showing that necrosis was not yet detectable. On the other hand, consumption of HF‐HCD enhanced the elevation in serum cTnI, indicating a greater myocardial injury than in rats fed with SD. Nevertheless, both cTnI and CK‐MB levels were lower in SYN‐administered hyperglycaemic rats fed with either diet, implicating the protective effect of synbiotic ingestion on limiting the extent of cardiac injury. These findings are verified by the STRI measurements, which are used for the haemodynamic evaluation and estimation of infarct area and the severity of myocardial damage following myocardial infarction (Schröder et al., 1995). In our study, when compared to the normoglycaemic control group, STRI measured following I/R was decreased in hyperglycaemic SD and HF‐HCD groups with a more dramatic decrease in hyperglycaemic HF‐HCD‐fed rats, indicating that cardiac injury is exaggerated in hyperglycaemic rats, and especially in HF‐HCD‐fed hyperglycaemic rats. On the other hand, STRI was elevated in SYN‐supplemented hyperglycaemic HF‐HCD‐fed rats, suggesting the facilitatory impact of SYN on cardiac recovery.

Blood pressure, another haemodynamic parameter, is a leading cause of cardiovascular events and leads to increased cardiac damage (Vlachopoulos et al., 2010). In our study, increased blood pressure in hyperglycaemic rats appears to contribute to the development of I/R‐induced cardiac damage, while SYN supplementation resulted in decreased blood pressure in hyperglycaemic rats fed with SD. In addition, cardiovascular risk factors that increase the severity of cardiac injury are correlated with increased levels of anxiety. In particular, the relationship between the development of hyperglycaemia and anxiety has been reported in a study (Gariepy et al., 2010). In our study, anxiety levels of hyperglycaemic rats fed with either diet were increased, while daily intake of SYN alleviated anxiety. On the other hand, serum corticosterone levels were similar in all rats fed with either diet. Previous studies have reported that levels of anxiety may not be correlated with the changes in cortisol concentrations (Chakraborty et al., 2020; Safari et al., 2020). In addition, it appears that the anxiety‐promoting factors, which altered the behavioural tests, were not sufficient enough to stimulate the hypothalamus–pituitary–adrenal axis.

Our findings demonstrate that SYN supplementation regulates the recovery of cardiac injury and depresses anxiety in conjunction with the suppression of the proinflammatory cytokine TNF‐α. In parallel with its critical role in inflammation and immune response TNF‐α, especially that produced by cardiac myocytes, can lead to heart diseases including biventricular dilatation, depressed ejection fractions, myocyte apoptosis, myocarditis, fibrosis and atrial thrombosis (Bryant et al., 1998; Moe et al., 2004). TNF‐α inhibits myocardial L‐type Ca^2+^ channels through type I TNF‐α receptors and prevents calcium ion entry into the myocardial cell, creating a negative inotropic effect on the myocardium and contributing to enhanced myocardial damage (Krown et al., 1995). On the other hand, TNF‐α is considered a key molecule in the development of hypertension (Fernandez‐Real et al., 2002; Huang et al., 2016), which may be caused by impaired endothelial Ca^2+^ signalling induced by vascular smooth muscle‐derived TNF‐α (Kuppusamy et al., 2022). In particular, mild increases in serum or tissue levels of TNF‐α increase blood pressure (Ramseyer & Garvin, 2013). Moreover, increased blood pressure may also increase TNF‐α production (Bergman et al., 1999). Significantly increased TNF‐α levels in hyperglycaemic rats under ischaemia–reperfusion conditions could have contributed to the severity of myocardial injury. It may be speculated that SYN supplementation may have acted as a TNF‐α inhibitor and ameliorated the increase in cardiac injury, in SD diet‐fed hyperglycaemic rats, which confirms the earlier suggestion that TNF‐α inhibition is a good strategy to prevent myocardial ischaemia and reperfusion injury (Pei et al., 2015).

IL‐6 produced by inflammatory cells as well as by viable myocytes surrounding the infarcted area after myocardial ischaemia also contributes to myocardial damage and fibrosis (Bennet et al., 2003; Welsh et al., 2008) and promotes infarct development by stimulating myocyte apoptosis (Gwechenberger et al., 1999). In addition, IL‐6 increases smooth muscle cell proliferation and migration, activation of macrophage‐monocytes, intima cell migration and LDL oxidation in coronary arteries, which lead to endothelial dysfunction (Feng et al., 2022). Increase in CVD risk factors increases IL‐6 production from cardiac fibroblast cells and induces myocardial fibrosis via the transforming growth factor‐β1 signalling pathway (Wang et al., 2016). These effects explain the contribution of increased IL‐6 levels to exaggeration of myocardial damage in hyperglycaemic rats under ischaemia–reperfusion conditions in our study. It was reported that a relationship exists between serum IL‐6 level and blood pressure (Fernandez‐Real et al., 2001), while IL‐6 inhibition has been reported to reduce the severity of cardiac damage and regulate blood pressure (Diego et al., 2024; Feng et al., 2022). Our results showed that having received long‐term SYN supplementation before I/R conditions abolished the elevation in serum IL‐6 level of hyperglycaemic rats and decreased the blood pressure of SD‐fed rats, but the elevated blood pressure in the HF‐HCD‐fed rats was not reduced by synbiotic intake. This suggests that suppression of elevated IL‐6 level by SYN is effective in reducing cardiac injury in HF‐HCD‐fed hyperglycaemic rats without any impact on the regulation of high blood pressure.

In our study, a correlation was observed between the increase in TNF‐α and IL‐6 levels and anxiety in hyperglycaemic rats on either diet. In another study, it was reported that suppression of the IL‐6 increment would decrease the level of anxiety (de Baumont et al., 2019). Based on the previous studies and our current findings, it can be suggested that synbiotics appear to have anxiolytic effects on anxiety by regulating TNF‐α and IL‐6 levels, which are effective in anxiety formation. In a recent systematic meta‐analysis, which evaluated the effects of synbiotics on anxiety by searching randomized controlled trials (Zhao et al., 2023), it was reported that synbiotics can reduce anxiety scores, but the mechanisms by which synbiotics improve anxiety remain unclear. These effects of synbiotic supplementation may arise from its regulatory role on the gut microbiota. The abundance of gram‐negative bacteria in the *Proteobacteria* phylum, which contain lipopolysaccharide (LPS) in their outer membrane, is accepted as an important marker of intestinal dysbiosis. LPS stimulates TNF‐α and IL‐6 production, inhibits nitric oxide production and thereby contributes to the development of low‐grade inflammation (Hirohashi & Morrison, 1996; Shi et al., 2002). Accordingly, increased TNF‐α and IL‐6 levels simulated by LPS would increase anxiety and enhance the severity of cardiovascular injury. However, long‐term synbiotic consumption decreased the abundance of *Proteobacteria* phylum, prevented the increase in proinflammatory cytokines and decreased anxiety and cardiac damage.

### 4.1 Limitations

In our study, rats following weaning were introduced to either a SD or a HF‐HCD and were induced with acute hyperglycaemia when they were juvenile. In reference to clinical relevance, further studies are needed in adult or aged rats exposed to longer periods of hyperglycaemic conditions. We studied the densities of four phyla which are majorly involved in dysbiosis; however, a more comprehensive microbiome analysis and data on the produced metabolites would also provide valuable information on the involvement of microbiota in the development of cardiovascular disease.

### 4.2 In conclusion

Addition of synbiotics to either a normal or a high‐fat high‐carbohydrate diet improves gut dysbiosis, reduces anxiety and cardiovascular risk factors, and alleviates myocardial ischaemia–reperfusion injury in hyperglycaemic rats.

## AUTHOR CONTRIBUTIONS

Each author has participated sufficiently in the conception, design (Erman Caner Bulut, Ebru Gürel Gürevin, Cihan Demirci Tansel, Berrak Ç. Yeğen), or acquisition (Erman Caner Bulut, Deniz Erol Kutucu, Savaş Üstünova, Mehmet Ağırbaşlı, Huri Dedeakayoğulları, Çağatay Tarhan, Ayşegül Kapucu) of data or analysis and interpretation (Erman Caner Bulut, Ebru Gürel Gürevin, Cihan Demirci Tansel, Berrak Ç. Yeğen, Mehmet Ağırbaşlı, Huri Dedeakayoğulları, Çağatay Tarhan) of data; in drafting the manuscript (Erman Caner Bulut, Deniz Erol Kutucu, Savaş Üstünova, Mehmet Ağırbaşlı, Huri Dedeakayoğulları, Çağatay Tarhan, Ayşegül Kapucu, Cihan Demirci Tansel) or revising it critically for important intellectual content (Ebru Gürel Gürevin, Berrak Ç. Yeğen). All authors have read and approved the final version of this manuscript and agree to be accountable for all aspects of the work in ensuring that questions related to the accuracy or integrity of any part of the work are appropriately investigated and resolved. All persons designated as authors qualify for authorship, and all those who qualify for authorship are listed.

## CONFLICT OF INTEREST

The authors declare that they have no conflict of interest.

## Data Availability

The data underlying our findings can be shared upon reasonable request directed to the corresponding author.
